# Recent advances of the nanocomposite hydrogel as a local drug delivery for diabetic ulcers

**DOI:** 10.3389/fbioe.2022.1039495

**Published:** 2022-10-04

**Authors:** Sen Tong, Qingyu Li, Qiaoyan Liu, Bo Song, Junzi Wu

**Affiliations:** ^1^ School of Basic Medical, Yunnan University of Chinese Medicine, Kunming, Yunnan, China; ^2^ School of Medicine, Jianghan University, Wuhan, China

**Keywords:** diabetic ulcer, nanocomposite hydrogel, nano delivery system, hydrogel, wet dressing

## Abstract

Diabetic ulcer is a serious complication of diabetes. Compared with that of healthy people, the skin of patients with a diabetic ulcer is more easily damaged and difficult to heal. Without early intervention, the disease will become increasingly serious, often leading to amputation or even death. Most current treatment methods cannot achieve a good wound healing effect. Numerous studies have shown that a nanocomposite hydrogel serves as an ideal drug delivery method to promote the healing of a diabetic ulcer because of its better drug loading capacity and stability. Nanocomposite hydrogels can be loaded with one or more drugs for application to chronic ulcer wounds to promote rapid wound healing. Therefore, this paper reviews the latest progress of delivery systems based on nanocomposite hydrogels in promoting diabetic ulcer healing. Through a review of the recent literature, we put forward the shortcomings and improvement strategies of nanocomposite hydrogels in the treatment of diabetic ulcers.

## Introduction

Diabetes mellitus is a chronic disease caused by the disorder of glucose metabolism. Long-term hyperglycaemia will increase the blood viscosity of patients, weaken the elasticity of blood vessels and weaken the local skin barrier function of patients, resulting in diabetic ulcers such as diabetic foot ulcers, leg ulcers, and buttock pressure ulcers (Fiordaliso et al., 2016; Yeom et al., 2016; Sun et al., 2022). For normal people, when the skin is damaged, the skin lesions can quickly and naturally heal after the four stages of haemostasis, inflammation, proliferation and maturation (Gurtner et al., 2008). For patients with diabetes, due to the imbalance of an inflammatory reaction around the ulcer wound, increased oxidative stress, bacterial infection, insufficient angiogenesis, hypoxia of wound tissue and other reasons, the wound cannot follow the normal and orderly repair process, such that delayed healing or no healing occurs, requiring long-term bed rest (Demiot et al., 2006; Leal et al., 2015; Hassan et al., 2019; Fadol et al., 2021; Singh et al., 2022).

At present, there are a variety of clinical methods to treat diabetic ulcers, mainly based on blood glucose control, timely dressing, debridement and prevention and control of infection (Zubair and Ahmad, 2019; Boyko et al., 2022). However, the effect of treatment is not ideal. Even with these standardised treatments, only a small proportion of diabetic ulcers heal after 12–20 weeks, and most patients require more advanced interventions (Frykberg et al., 2020). According to past clinical experience, the traditional dry dressing can keep the wound dry and absorb part of the wound exudate (Jeffcoate et al., 2009). However, they cannot maintain the temperature and humidity of the wound and cannot protect the wound from bacterial invasion. Moreover, the traditional dressing readily adheres to the new granulation tissue of the wound, causing secondary injury when the dressing is changed (Hilton et al., 2004). These reasons contribute to the limitations of dry dressings in clinical practice.

With the continuous development of biomedical materials and in-depth studies of wound healing theory, investigators found that a clean and humid environment is more conducive to wound healing. Therefore, wet wound dressings, particularly hydrogels have attracted attention (Sirousazar et al., 2011; Zhang X. et al., 2019). A hydrogel is a water-insoluble gelatinous material. Compared with traditional dry dressings, hydrogels can create a healing environment faithfully reflecting the physiological state, provide extra moisture for dry and scabby wounds, moisten exposed tissues, and nerve endings and reduce pain when the dressing is changed (Li Y. et al., 2017; Zhou et al., 2018). Hydrogels can retain the active substances in the exudate of patients, promote cell division and migration and promote rapid wound healing. However, hydrogels have limitations such as low mechanical strength, sudden drug release, susceptibility to degradation and high storage requirements (Zhu et al., 2018; Ma et al., 2020; Luo Y. et al., 2022). Once dehydrated, the appearance and properties of hydrogels will be greatly affected, which limits their wide application in the biomedical field.

To overcome these limitations, researchers worldwide compete to conduct studies aimed to improve the mechanical properties of hydrogels. With the continuous development of nanotechnology, researchers have found that nanocomposite hydrogels can be prepared by introducing nanostructured particles into the hydrogel network through chemical bonding or physical adsorption. These methods can improve the mechanical strength of hydrogels and impart some new properties to hydrogels (Li et al., 2018; Chen et al., 2019; Shin et al., 2019; Abdollahi et al., 2021). Specifically, a nanocomposite hydrogel is a three-dimensional network formed by physical or chemical crosslinking of natural, synthetic hydrophilic polymers or both (Wei et al., 2019). It has abundant pores and good hydrophilicity, which is conducive to gas exchange and can maintain the fluid balance at the wound site (Xavier et al., 2015; Zhai et al., 2018). Its porous structure can simulate the structure and function of the extracellular matrix and promote cell migration, proliferation and maturation (Liu et al., 2021). Briefly, a nanocomposite hydrogel has the advantages of a hydrogel mentioned above and the excellent characteristics of nanoparticles. Furthermore, nanocomposite hydrogels are a well-characterised treatment method that promotes the healing of diabetic ulcers and provides a new strategy for the treatment of diabetic ulcers.

In particular, nanocomposite hydrogels are advantageous, because they can be loaded with small-molecule drugs, biomacromolecules, inorganic nanoparticles and other active substances that enhance the biological activity of nanocomposite hydrogels and further expand their application to wound management (Alvarez et al., 2014; Wu et al., 2019; Yang et al., 2021; Massironi et al., 2022; Rao et al., 2022). When a nanocomposite hydrogel is used as drug delivery system carrier, it can reach the lesion through active or passive transport, which is suitable for drug delivery to the skin, oral mucosa, eye and some gastrointestinal mucosa (Sapino et al., 2019; Wróblewska et al., 2020; Cheng et al., 2021; Li et al., 2022a). Compared with other types of nano carriers, nanocomposite hydrogels are similar to natural extracellular matrices, with good water dispersion and are easier to penetrate human skin, achieve the locally sustained and on demand release of drugs and form a physical barrier to create a clean and moist healing environment for the repair of diabetic ulcer (Lou et al., 2021; Girija et al., 2022). Compared with ordinary hydrogels, nanocomposite hydrogels have better mechanical properties, a longer swelling process and higher drug loading capacity (Qiu et al., 2021).

Nanohydrogels have attracted increasing attention because of their great potential for use as effective drug delivery systems. This review focuses on the promotion of ulcer healing in animal models of diabetes or patients with diabetes through loading small-molecule drugs, protein drugs, silver nanoparticles and other substances. Furthermore, we evaluated the challenges to translating these nanocomposite hydrogels to the clinic.

## Application of a nanocomposite hydrogel delivery system to diabetic ulcers

### Treatment of diabetic ulcers with nanocomposite hydrogel loaded with small-molecule drugs

There are many advantages in using nanocomposite hydrogel as a carrier of small-molecule drugs: 1) It can protect drugs and improve their stability (Qi et al., 2020). 2) It can increase the solubility of the drug (Bang et al., 2019). 3) The effective treatment time was increased by sustained release of drugs (Luo F. Q. et al., 2022). 4) It can reduce the side effects of drugs (Yang X. et al., 2022). 5) The drug is delivered to the wound surface of a diabetic ulcer in a non-invasive or minimally invasive way to minimise tissue damage (Lei and Fan, 2022). At present, most patients with diabetic ulcers can be treated with small-molecule drugs during the early stage of disease. Combined with a patients’ status, the development of a personalised treatment programme can achieve better treatment efficacy.

**FIGURE 1:**
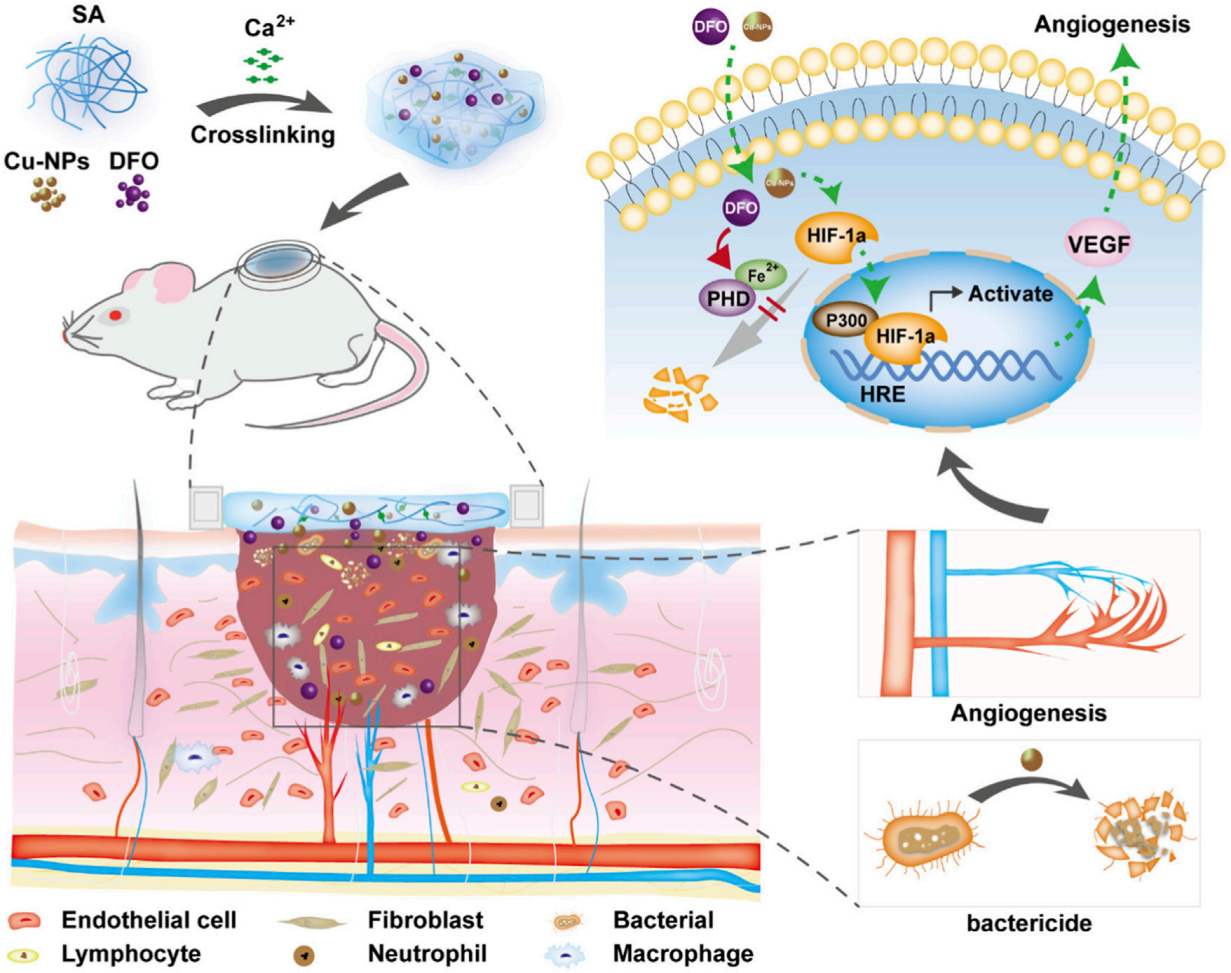
Schematic diagram of the strategy for the treatment of diabetic wound healing using bioactive hydrogel composites containing deferoxamine and copper nanoparticles. Reproduced from (Li et al., 2022b), with permission from Elsevier.

Deferoxamine (DFO), a small-molecule drug approved by the Food and Drug Administration (FDA) of the United States, has been used to induce angiogenesis in bone and skin regeneration (Lintel et al., 2022). DFO can induce angiogenesis and is used in the treatment of diabetic foot ulcers (Ram et al., 2015). It has been found that DFO-laden silk nanofiber hydraulics provided a sustained release of DFO for more than 40 days and is used to treat diabetic wounds, which can effectively regulate inflammation, regulate the migration and gene expression of endothelial cells, improve the deposition of the extracellular matrix and accelerate the healing of diabetic ulcers (Ding et al., 2021). Another study found that a sodium alginate composite hydrogel containing DFO and copper nanoparticles (Cu NPs) was prepared using a calcium ion crosslinking method, which conferred beneficial effects on diabetic ulcers (Li et al., 2022b). The hydrogel has synergistic effects on the proliferation, migration and angiogenesis of human umbilical vein endothelial cells *in vitro*. Further, the hydrogel upregulates vascular endothelial growth factor (VEGF) and hypoxia-inducible factor-1α, which can accelerate the healing of a diabetic ulcer (Figure 1).

4-Hydroxy-3-methoxycinnamic acid (ferulic acid) is a natural antioxidant with antibacterial activity. It can reduce blood glucose levels, scavenge free radicals, promote angiogenesis and so on (Karamać et al., 2017). It was found that ferulic acid was encapsulated into nanoparticles using the nano precipitation method, and then Carbopol 980 was added to form a ferulic acid nanohydrogel (Bairagi et al., 2018). The results of subsequent animal experiments show that a ferulic acid nanohydrogel significantly increases the content of hydroxyproline, promotes collagen deposition and promotes tissue repair and wound healing in diabetic rats.

3-Methyl-1-phenyl-2-pyrazolin-5-one (edaravone) strongly scavenges free radicals and promotes wound healing, although its poor stability and solubility hinders its local application (Fujisawa and Yamamoto, 2016; Parikh et al., 2016; Tamer et al., 2018). It was found that the sustained release of edaravone could be effectively achieved by loading edaravone nanoparticles into a sodium alginate hydrogel (Fan et al., 2019). Edaravone encapsulated in a nanocomposite hydrogel accelerates wound healing faster than free edaravone. This study further found that edaravone has different scavenging effects on reactive oxygen species. A low dose of edaravone benefits wound repair, while a high dose hinders wound healing.

**FIGURE 2:**
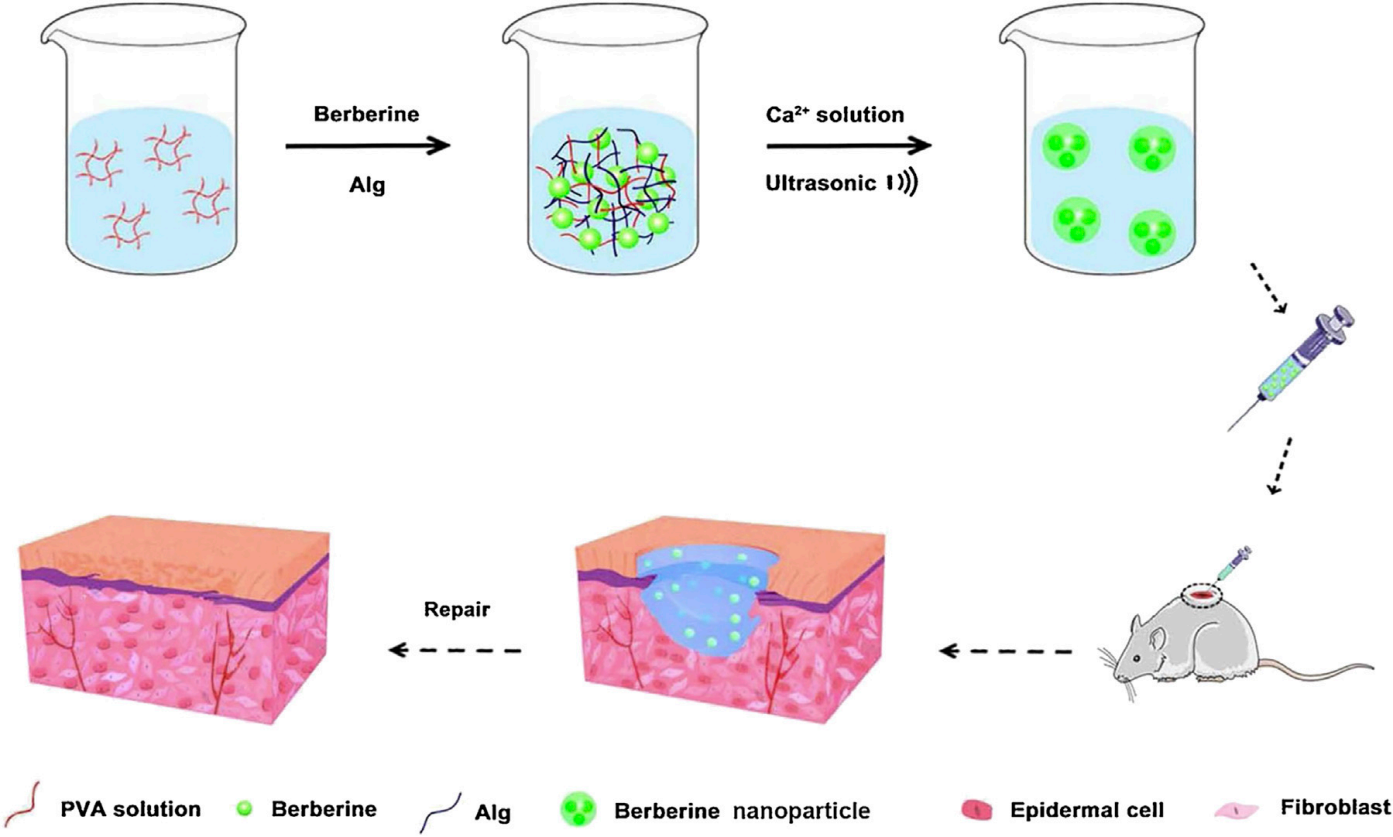
Preparation of a berberine nanohydrogel and its application to skin wound healing of diabetic rats. Reproduced from (Zhang et al., 2020), with permission from Elsevier.

Berberine has anti-inflammatory, antibacterial and hypoglycaemic effects, promoting the healing of diabetic ulcers (Dou et al., 2021; Maity et al., 2022; Zhang et al., 2022). However, owing to the low water solubility and lipid solubility of berberine, its oral bioavailability is low, which seriously limits the development and application of berberine as a pharmaceutical (Sahibzada et al., 2018). It has been found that a nanohydrogel can be used as a carrier for sustained release of berberine (Zhang et al., 2020). Berberine encapsulated in a nanohydrogel has stronger water holding capacity than ordinary berberine hydrogel and can promote the migration and proliferation of epidermal cells required for wound repair. Furthermore, berberine nanohydrogel could inhibit the expression of nuclear factor kappa-B, tumor necrosis factor-α, and interleukin 6 by activating silent information regulator 1 and increase the expression of VEGF and platelet endothelial cell adhesion molecule-1, which finally promoted the healing of diabetic ulcers (Figure 2).

7-Glucuronic acid 5,6-dihydroxyflavone (baicalin) can promote the expression of epidermal growth factor and VEGF and then enhance the regenerative ability of epidermal cells, endothelial cells, and fibrotic tissue, to induce wound healing (Zhang et al., 2011; Mao et al., 2021). It has been found that a baicalin-loaded nanohydrogel has suitable viscosity, good skin retention and good biocompatibility, which can antagonise the cytotoxic effects of hydrogen peroxide, regulate the inflammatory process and promote the healing of chronic wounds (Manconi et al., 2018).

**FIGURE 3:**
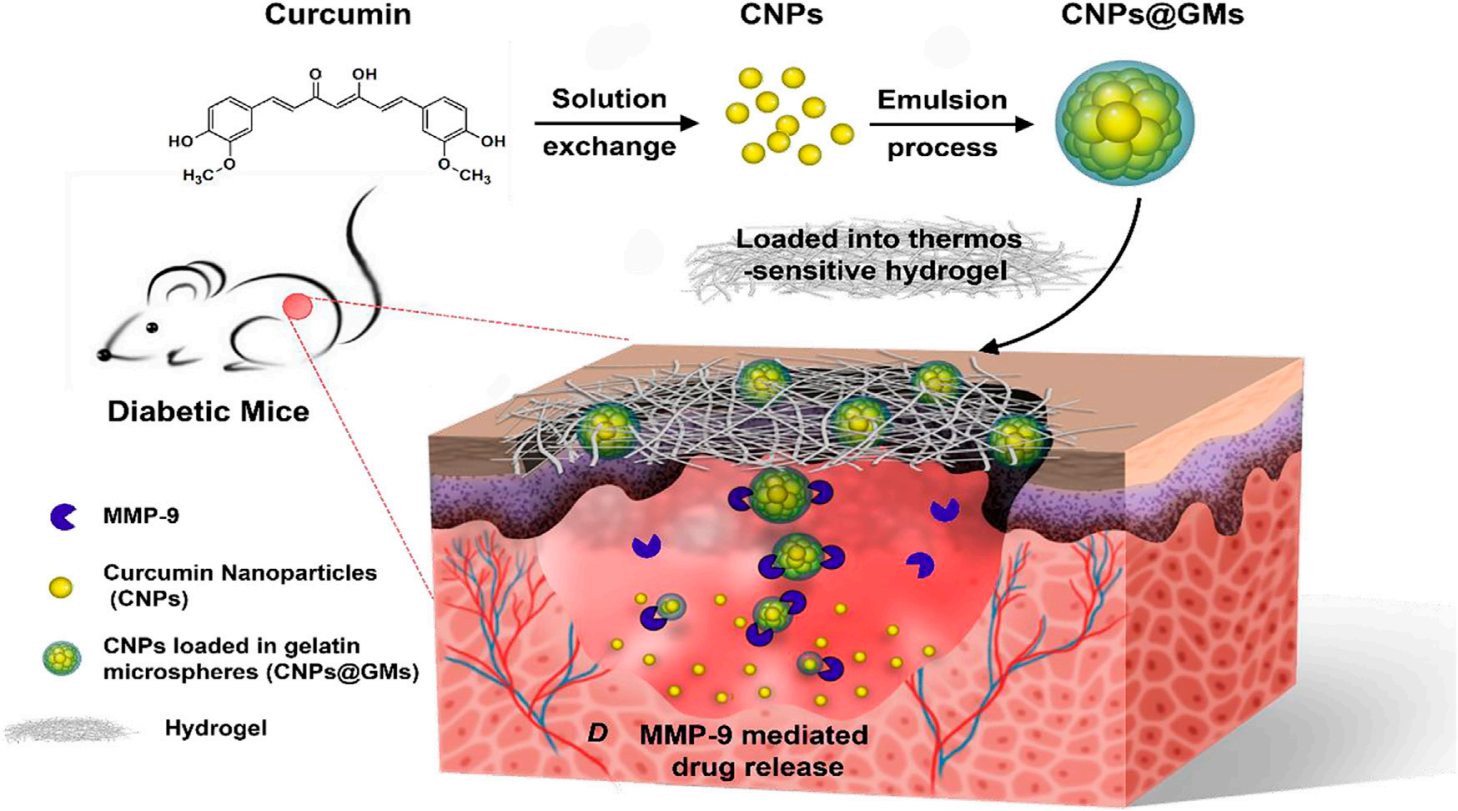
The preparation of curcumin nanoparticles/gelatin-microsphere hydrogel and the drug release process in the wound beds of diabetic mice. Reproduced from (Liu et al., 2018), with permission from American Chemical Society.

Curcumin has been shown to promote wound healing (Jose et al., 2022). However, its water solubility and skin permeability are poor, which limits its local application (Naz and Ahmad, 2015). A recent study found that curcumin-loaded nanocomposite hydrogels can prolong the local treatment time of curcumin compared with ordinary curcumin hydrogels (Kamar et al., 2019). Slow release of curcumin can promote the healing of diabetic ulcers by improving the wound closure rate, accelerating the formation of granulation tissue and the deposition of collagen deposition, thus enhancing the expression of VEGF and Aquaporin 3. Other studies have found that gelatin microspheres containing curcumin nanoparticles can ensure the stable release of curcumin in the wound surface, significantly improve the antioxidant effect and migration-inducing ability of curcumin and promote skin wound healing of diabetic mice (Liu et al., 2018) (Figure 3).

Previous studies have confirmed that quercetin or oleic acid alone can promote wound healing (Rodrigues et al., 2016; Jangde et al., 2018). A recent study found that the nanohydrogel prepared using quercetin and oleic acid can reduce the pain of patients with diabetic ulcers, improve the viscoelasticity of tissue and significantly shorten wound healing time (Gallelli et al., 2020).

### Treatment of diabetic ulcers with nanocomposite hydrogel loaded with protein

At present, most studies focus on the use of protein hormones and growth factors to promote the healing of diabetic ulcers (Laiva et al., 2021; Oliveira et al., 2021; Mohammadi Tofigh and Tajik, 2022). Compared with small-molecule drugs, protein drugs have become a hot field of new drug development because of their high activity, high specificity, low physiological toxicity and good biocompatibility. However, the unique molecular structure and biochemical characteristics rapidly inactivate protein drugs in complex biological environments, which limits their application to biomedicine (Yu Y. et al., 2021). It has been shown that nanocomposite hydrogels can encapsulate protein drugs in their internal network structure, thus protecting them from the external environment (Li M. et al., 2017; Wang Z. et al., 2019; Zhou et al., 2022).

Proteins are important nutrients for the proliferation and remodelling of wound healing (Cheng et al., 2020; Smith et al., 2022). Proteins can promote the formation of capillaries, the proliferation of fibroblasts and the synthesis of collagen and improve the function of the immune system. Therefore, exogenous protein supplementation can promote wound healing and tissue repair (Kim et al., 2019; Lapi et al., 2021; Yang P. et al., 2022).

Insulin is a physiological hypoglycaemic agent, which is commonly used in the treatment of diabetes. Recent studies have found that local use of insulin can treat diabetic ulcer (Zhang and Lv, 2016; Vatankhah et al., 2017; Bhettani et al., 2020). Both free insulin and nano insulin encapsulated in a hydrogel can improve wound healing, although insulin in a nanocomposite hydrogel has a better therapeutic effect (Abdelkader et al., 2018). Furthermore, insulin can reduce inflammation, increase angiogenesis, induce the formation of granulation tissue, reconstruct the epidermis and completely deposit collagen.

**FIGURE 4:**
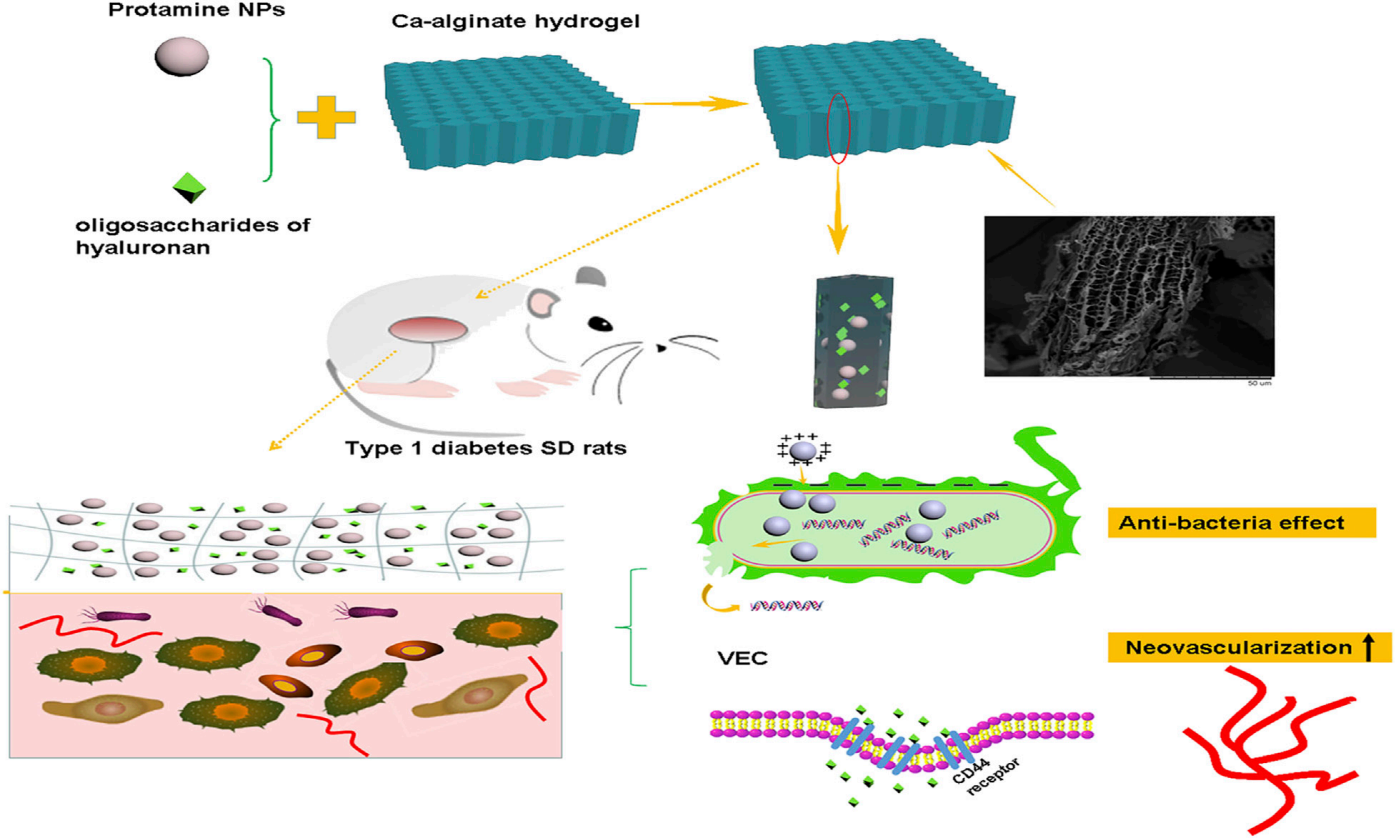
Application of a calcium alginate gel loaded with protamine nanoparticles and hyaluronan oligosaccharides in skin wound healing of type 1 diabetic rats. Reproduced from (Wang T. et al., 2019), with permission from Springer Nature.

Based on the hypothesis that regulating antibacterial and neovascularisation activities promotes diabetic wound healing, a pH-responsive calcium alginate hydrogel was reported, which loaded protamine nanoparticles and hyaluronic acid oligosaccharides, showing good homogeneity and biocompatibility (Wang T. et al., 2019; Guo et al., 2022; Liu et al., 2022). Protamine nanoparticles act as a cationic antibacterial peptide against various bacteria by disrupting their cell membranes. The addition of hyaluronic acid oligosaccharides promoted the migration of human umbilical vein endothelial cells *in vitro* and the secretion of VEGF. Protamine nanoparticles and hyaluronic acid oligosaccharides synergise to promote wound healing (Figure 4).

Silk fibroin is mainly composed of non-polar amino acids. It has good biocompatibility and no toxicity. It can promote cell proliferation and differentiation *in vivo* and *in vitro*, which is conducive to wound healing (Park Y. R. et al., 2018; Zhang Y. et al., 2019). However, it is difficult to prepare single-network silk fibroin hydrogels for the treatment of diabetic ulcers because of the secondary structural transformation of silk fibroin, which often produces a large β-sheet domain that greatly increases the brittleness of hydrogels (Xu et al., 2021). It has been found that natural biomaterials such as silk fibroin, carboxymethyl cellulose and manganese dioxide nanoflakes can be used to prepare injectable nanocomposite hydrogels, which can provide a moist wound healing environment, relieve inflammation, promote angiogenesis and matrix remodelling and adapt to the irregular wound shape of patients with diabetic ulcer (Pu et al., 2022). Numerous studies have shown that under the condition of high oxidative stress in patients with diabetes, the overexpression of matrix metalloproteinases impairs the formation of granulation tissue and the regeneration of the extracellular matrix (Wu et al., 2016). The above hydrogel can effectively neutralise excessive matrix metalloproteinases, reduce the level of inflammation and promote the integration of extracellular matrix and angiogenesis. The uniform dispersion of MnO_2_ nanosheets endows hydrogels with outstanding reactive oxygen species scavenging ability that alleviates oxidative stress and produces sustained O_2_ to promote angiogenesis.

In a study, an inflammatory memory peptide was extracted from absent in melanoma 2 to prepare a nanopeptide hydrogel (Cheng et al., 2018). Subsequently, *Akk* *ermansia muciniphila*, a member of the intestinal microbiota with hypoglycaemic effect, was inoculated into the hydrogel. After subcutaneous injection, the hydrogel was delivered to the diabetic ischaemic ulcer tissue. It was found that the hydrogel could promote angiogenesis, regulate the immune response and reduce local glucose levels to obviously promote the healing of diabetic ulcers. Components of protein hydrolysates such as glutamic acid, aspartic acid and glycine can promote wound healing (Corsetti et al., 2017; Hung et al., 2019). A paper reports a multifunctional nanocomposite hydrogel with excellent tensile and compressive properties, rapid recovery ability, antibacterial activity and coagulation ability (Tavakoli et al., 2020). The presence of l-glutamic acid accelerates the migration of wound cells and the formation of a scar in rats with diabetic ulcer and promotes the synthesis of collagen on the wound surface.

Many studies have confirmed that wound healing is a complex process involving a variety of cells and cytokines (Park K. H. et al., 2018; Chen et al., 2018; Certelli et al., 2021; Schirmer et al., 2021). Patients with diabetes experience long-term wound healing because of the absence of local growth factors with diminished activity (such as the glycosylation of growth factor). Therefore, exogenous growth factors may promote wound healing.

Among growth factors, Epidermal Growth Factor (EGF) is a low-molecular-weight peptide composed of 53 amino acids (Guo et al., 2020). Previous studies have shown that EGF can greatly promote the proliferation and movement of keratinocytes and fibroblasts, granulation tissue formation, and extracellular matrix synthesis. Exogenous recombinant human EGF (rhEGF) is one of the most widely used growth factors in the treatment of diabetic ulcer (Tsang et al., 2003; Jeong et al., 2020; de Oliveira et al., 2022). A study reported that a chitosan-based composite hydrogel can be produced by compounding EGF-coated nanoparticles with silver ions using nanocapsule technology (Lee et al., 2021). The hydrogel can continuously release EGF and Ag^+^, has obvious bactericidal effect and has good hydration effect, which makes it very suitable for wound exudation environment. Compared with other methods of treating diabetic ulcers, the hydrogel resulted in rapid collagen deposition, reduced inflammation, and faster wound healing in rats with diabetic ulcers. Another study reported that a chitosan-based nanocomposite hydrogel could be developed through nanotechnology, which encapsulates EGF nanoparticles, perfluorocarbon and polyhexamethylene biguanide (Lee and Lin, 2022). The hydrogel continuously released EGF and polyhexamethylene biguanide, which had antibacterial, anti-inflammatory, oxygen transport and enhanced cell growth activities. These functions allow the skin tissue integrity and function of diabetic rats to rapidly recover. In addition, a study reported that a polymer coupled to recombinant human epidermal growth factor incorporated into chitosan-based hydrogels, which retains the biological activity of EGF and confers better resistance to proteolysis (Hajimiri et al., 2016). It can promote the proliferation of fibroblasts *in vitro* and promote wound healing in diabetic rats.

Platelet-derived growth factor (PDGF), known as “trauma factor”, is mainly stored in platelet alpha granules (Walsh and Poole, 2017). When the body is injured, epithelial cells, endothelial cells, macrophages and immune cells secrete PDGF (Yang et al., 2020). They are directly or indirectly involved in inflammatory reaction, tissue and cell differentiation and proliferation of wound repair (Das et al., 2016; Jian et al., 2022). In 1997, the FDA approved *Becaplermin* containing a human recombinant platelet-derived growth factor BB (PDGF-BB) gel for the treatment of diabetic foot ulcers. A recent study reported that PDGF-BB encapsulated with a self-assembled peptide (RADA 16-I) can create a kind of nanohydrogel with good biocompatibility and biodegradability, which allows continuous delivery of PDGF-BB and destroys the bacterial biofilm (Santhini et al., 2022). Compared with the control group and untreated animals, the levels of hydroxyproline and ascorbic acid in *Rattus norvegicus* treated with the PDGF-BB nanohydrogel were significantly increased, which confirms the role of PDGF-BB nanohydrogel in promoting angiogenesis and wound healing.

Stromal cell-derived factor-1α (SDF-1α) is a key chemokine involved in the regulation of tissue and organ injury and repair with strong chemotaxis on diverse inflammatory and mesenchymal stem cells (Li et al., 2016). SDF-1α can promote angiogenesis and wound healing by inducing the migration of endothelial progenitor cells. After the expression of SDF-1α is inhibited, wound neovascularisation is significantly reduced, the inflammatory reaction is intensified and the wound healing rate is significantly reduced. To protect SDF-1α, Yu et al. (2020) prepared a kind of nanogel-loaded liposome that stimulates the recruitment of bone marrow mesenchymal stem cells, secretes cytokines, regulates the phenotypes of other effector cells and effectively promotes wound closure and tissue regeneration. Other studies have found that these nanocomposite hydrogels containing SDF-1α could influence macrophage phenotype and promote skin tissue regeneration in diabetic mice (Yu J. R. et al., 2021).

Moreover, combining small-molecule drugs with growth factors may be a promising method to promote wound healing. The curcumin and EGF modified by nanotechnology were encapsulated in hydrogels, which could release curcumin and EGF on demand and synchronise with wound healing. Specifically, curcumin is rapidly and continuously released during the early stage of wound healing to alleviate inflammation and oxidative stress, while EGF is relatively slow-acting and sustains late proliferation and extracellular matrix remodelling (Li Y. et al., 2021; Hu et al., 2021). A similar combination has been verified using nano lipid carriers (Lee et al., 2020).

### Treatment of diabetic ulcers with nanocomposite hydrogel loaded with silver nanoparticles

Bacterial infection is an important factor that hinders wound healing of diabetic ulcers (Kishibe et al., 2018; Zhang R. et al., 2021; Roy et al., 2022). The decomposition products of diabetic ulcer skin tissue and the persistent high-glucose environment provide an excellent culture medium for the growth and reproduction of bacteria. A large number of bacteria enter the human body through the wound and go deep into the tissue, leading to sepsis and even death (Shaheen et al., 2021; Xie et al., 2022). Early, active and effective control can prevent chronic infection and accelerate wound healing (Han and Kang, 2013).

Most metal nanoparticles possess antibacterial effects, among which that of silver nanoparticles is the strongest. Silver nanoparticles have excellent antibacterial properties through controlling the slow release of silver ions from their surface (Devi et al., 2017; Shankar and Rhim, 2017; Li M. et al., 2021; Haghniaz et al., 2021). In addition to antibacterial activity, silver nanoparticles have been observed to be anti-inflammatory, induce apoptosis of neutrophils, reduce the activity of matrix metalloproteinases, accelerate wound healing and reduce the formation of a scar (Wong et al., 2009; Sivakumar et al., 2017; Shehabeldine et al., 2022).

**FIGURE 5:**
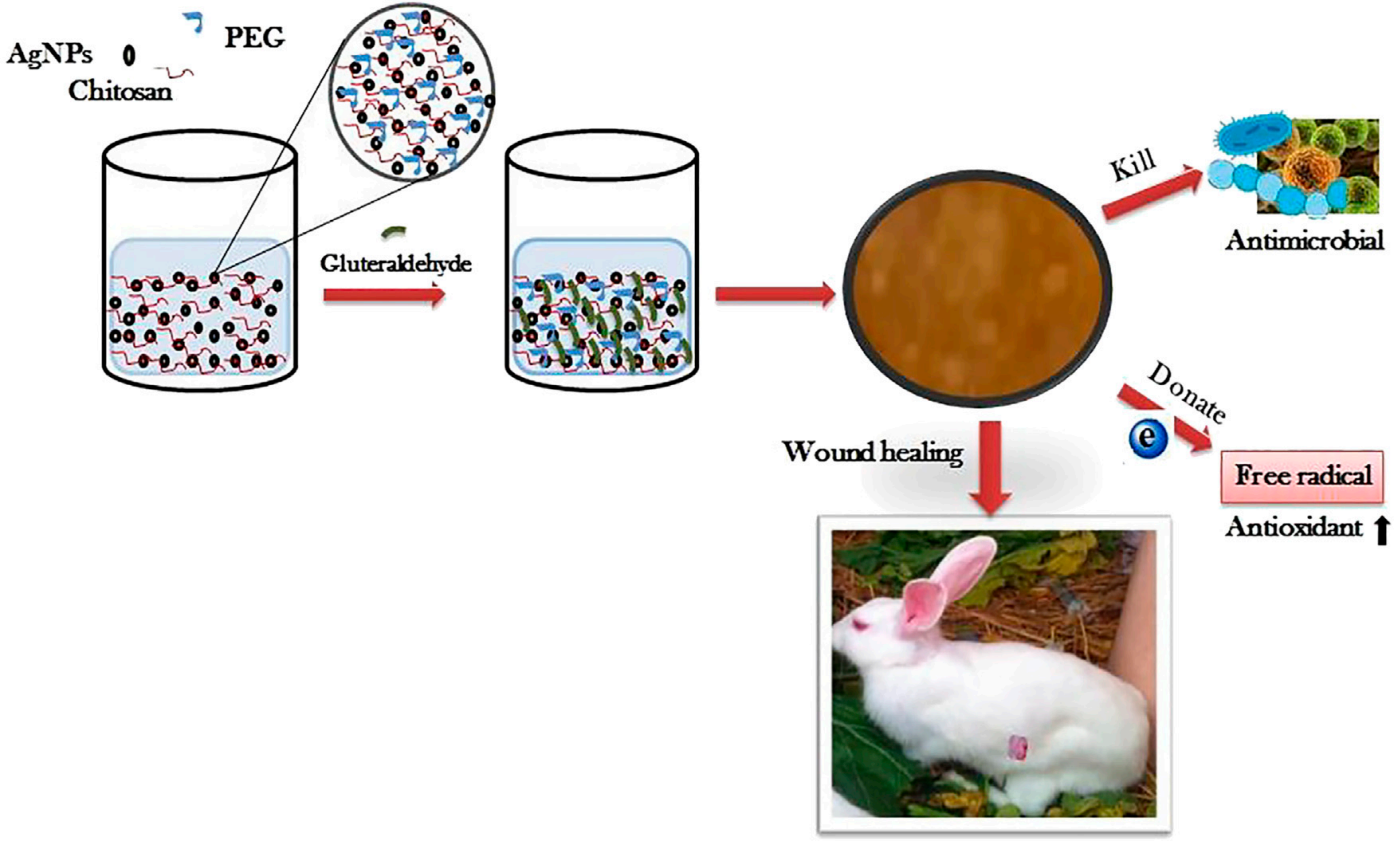
The preparation process of Silver nanoparticle impregnated chitosan-PEG hydrogel and its therapeutic effect on diabetic rabbit wounds. Reproduced from (Masood et al., 2019), with permission from Elsevier.

Recent studies have shown that bamboo cellulose nanocrystals can be impregnated with silver nanoparticles to obtain inexpensive nanocomposite hydrogels (Singla et al., 2017). The levels of pro-inflammatory cytokines IL-6 and TNF-α were significantly decreased, and the expression of collagen and growth factors (FGF, PDGF, VEGF) were significantly increased in mice with diabetic ulcers treated with topical application of this hydrogel. In addition, re-epithelialisation, angiogenesis and collagen deposition were improved, and wound healing was accelerated. Similarly, a type of chitosan polyethylene glycol hydrogel impregnated with silver nanoparticles was recently reported (Masood et al., 2019). Compared with the blank chitosan polyethylene glycol hydrogel, the silver nanoparticle-impregnated hydrogel has higher porosity, higher expansion and stronger antibacterial and antioxidant properties *in vitro*. The chitosan polyethylene glycol hydrogel impregnated with silver nanoparticles can slowly and continuously releases silver nanoparticles within 7 days, can accelerate the re-epithelisation and collagen deposition of diabetic wounds of rabbits and have better wound healing ability (Figure 5).

Recently, it has been reported that a novel antifouling and anti-infective hydrogel can be prepared by mixing silver nanoparticles with chitosan and dextran. After treatment with this hydrogel, the wounds of diabetic SD rats contracted rapidly, and the expression levels of CD68^+^ and CD3^+^ were upregulated (Shi et al., 2019). It is suggested that the hydrogel can promote the activation of immune cells and accelerate wound healing by promoting fibroblast migration, granulation tissue formation and angiogenesis. For example, Huang et al. (2017) synthesised nanocomposite hydrogels containing silver nanoparticles, showing high water content and antifouling and antibacterial properties. They achieve good absorption of exudates and are easily removed from wounds. The embedded silver nanoparticles continuously release Ag^+^ and eliminate adsorbed bacteria by interacting with sulfur-containing proteins on bacterial membrane. Furthermore, Ragothaman et al. (2021) have fabricated a nanohydrogel system coated with silver nanoparticles and melatonin, in which silver nanoparticles act as antibacterials and melatonin as an antioxidant and anti-inflammatory agent. The synergistic effect of the two drugs results in rapid tissue regeneration, collagen deposition and angiogenesis in the wound site of Wistar albino rats.

However, the latest research shows that silver nanoparticles readily agglomerate, and high concentration of Ag^+^ will have adverse side effects on human cells and tissues (Rana and Kalaichelvan, 2013). Based on this, a nanohydrogel loaded with functional Ag_2_S quantum dots was reported (Du et al., 2022). It can improve the bacterial clearance rate, increase the collagen coverage area and upregulate the expression of VEGF. It shows high biocompatibility and serves a new way for the treatment of infectious diabetic ulcers.

### Other considerations

**FIGURE 6:**
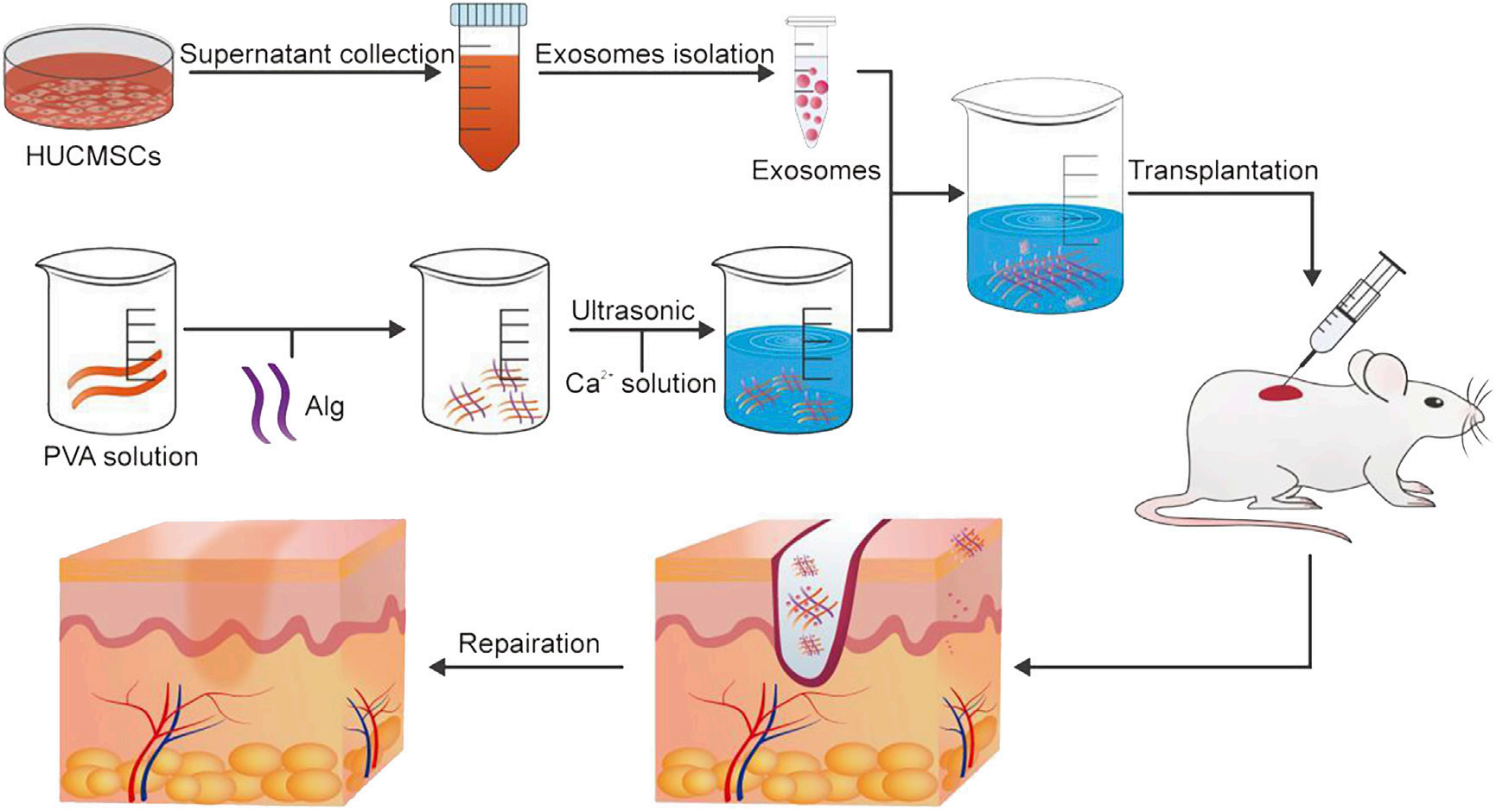
The preparation method of exosomes and the application of exosomes in wound healing. Reproduced from (Zhang Y. et al., 2021), with permission from Elsevier.

Exosomes are lipid bilayer extracellular vesicles secreted by various cells, which contain a series of bioactive substances such as proteins, lipids, RNA and DNA. They can promote angiogenesis, stimulate collagen deposition, inhibit inflammation and accelerate wound healing (Chen et al., 2021; Zhao et al., 2021; Wang et al., 2022). Previous studies have shown that the lack of exosomes may lead to delayed wound healing in patients with diabetic ulcers. It was found that the bioactive scaffold was prepared by wrapping exosomes from human umbilical cord mesenchymal stem cells in polyvinyl alcohol/alginate nanohydrogels (Zhang Y. et al., 2021). It has good biocompatibility and can effectively load exosomes to make them active. The hydrogel can promote the expression of smooth muscle actin, scavenger receptor class B type 1, platelet endothelial cell adhesion molecule-1 and VEGF by activating the ERK1/2 pathway and accelerate the wound healing of diabetic ulcers of rats. Furthermore, exosomes encapsulated in the nanohydrogel could promote the wound healing, proliferation, migration and angiogenesis of human umbilical cord mesenchymal stem cells in rats with diabetic ulcers more quickly than normally injected exosomes (Figure 6).

Recent studies have shown that platelet-rich plasma comprising numerous cell growth factors can accelerate wound healing by regulating cell proliferation (Menchisheva et al., 2019; Devereaux et al., 2020; Qian et al., 2020). However, most of the platelet-rich plasma used in the clinic is liquid, which cannot be fixed in the local area, and the growth factors in platelet-rich plasma is released too fast to maintain the long-term effective concentration. Recently, it has been reported that the plasma-rich gel is an effective carrier to protect platelet growth (Li et al., 2022a). *In vivo* studies showed that the hydrogel significantly promotes the healing of full-thickness skin wounds by enhancing granulation tissue formation, promoting collagen deposition and accelerating re-epithelialisation and neovascularisation.

Oxygen is the most important nutrient for cell survival. Insufficient oxygen delivery prevents cell migration and angiogenesis and reduces cell growth and differentiation, thereby delaying wound healing (Shiekh et al., 2020; Tu et al., 2022). Recently, a new technology is reported, which can transform the traditional gel dressing by adding freeze-dried nano-oxygen-containing powder to obtain a nano-sized oxygen-containing gel, which can deliver dissolved oxygen to the local wound surface (Yang Z. et al., 2022). The hydrogel promotes cell migration, angiogenesis and cell viability by antagonising the hypoxic environment of epithelial cells, endothelial cells and fibroblasts. Animal experiments showed that a nano-oxygen-containing gel has substantial effects on wound healing and flap survival in diabetic mice.

Increasing evidence shows that mesenchymal stem cells can promote wound healing of diabetic ulcers by promoting cell proliferation and differentiation and extracellular matrix synthesis, releasing growth factors and promoting angiogenesis (Han et al., 2019; Lv et al., 2020; Wang et al., 2022). Recently, it has been reported that nanopeptide hydrogels can improve the survival and proliferation efficiency of mesenchymal stem cells and enhance their differentiation potential and secretory activity (Xue et al., 2022). The hydrogel can regulate inflammatory reactions by downregulating inflammatory factors and upregulating VEGF to promote angiogenesis, thus accelerating the healing of diabetic ulcers.

Recently, researchers have found that proteins in egg white play an important role in wound healing (Ge et al., 2021). Based on this, montmorillonite/polyvinyl alcohol nanocomposite hydrogels containing egg white were prepared using a cyclic freeze-thaw method (Jahani-Javanmardi et al., 2016). Such hydrogels are transparent, and the equilibrium water content and gas exchange rate are very close to those of human skin. Moreover, it is suitable for dry diabetic ulcer wounds.

## Discussion

Diabetic skin ulcers are a serious complication caused by the internal environmental changes and the local pathological changes of skin caused by diabetes. Many studies have shown that a nanocomposite hydrogel is an ideal local drug delivery method to promote the healing of diabetic ulcers and has achieved satisfactory therapeutic effects. Nanocomposite hydrogels have the advantages of small particle size, high water content, long drug released property and good biocompatibility and biodegradability. They can also be used for the delivery of small-molecule drugs, protein drugs and silver nanoparticles to promote the healing of diabetic ulcers. In conclusion, nanocomposite hydrogels are expected to serve as a new dressing for large-scale clinical treatment of diabetic ulcer patients.

However, there are still problems to be solved. For example, most nanocomposite hydrogels are in the early stage of experimental or clinical development, and there is a lack of large-scale clinical studies to establish efficacy and safety. The preparation of some hydrogels is complex, which makes it difficult to widely apply them to the clinic. To reduce the adverse reactions in clinical treatment, it is necessary to accurately control a series of properties of nanocomposite hydrogels, such as crosslinking degree, porosity, swelling, mechanical properties, cell adhesion and permeability and to simulate the extracellular matrix microenvironment as much as possible to maintain the characteristics and activity of each component. Furthermore, it is important to determine the optimal moisture removal rate of the composite hydrogel and to determine the optimal moisture content of the composite hydrogel for patients with diabetes. It can be predicted that our research on nanocomposite hydrogel materials will be more detailed, and the preparation methods will be more advanced. More new nanocomposite hydrogel carriers will be developed and applied to clinical drug delivery, which will benefit more patients with chronic ulcers.
